# Effects of socioeconomic status on enrollment in clinical trials for cancer: A systematic review

**DOI:** 10.1002/cam4.6905

**Published:** 2024-01-03

**Authors:** Maja Wichhart Donzo, Grace Nguyen, John K. Nemeth, Maryanna S. Owoc, Leila J. Mady, Amy Y. Chen, Nicole C. Schmitt

**Affiliations:** ^1^ Department of Otolaryngology – Head and Neck Surgery Emory University School of Medicine Atlanta Georgia USA; ^2^ The Winship Cancer Institute at Emory University Atlanta Georgia USA; ^3^ Woodruff Health Sciences Center Library Emory University Atlanta Georgia USA; ^4^ University of Pittsburgh School of Medicine Pittsburgh Pennsylvania USA; ^5^ Department of Otolaryngology – Head and Neck Surgery Johns Hopkins University School of Medicine Baltimore Maryland USA

**Keywords:** cancer, clinical trials, disparity, socioeconomic status

## Abstract

**Background:**

To achieve equitable access to cancer clinical trials (CCTs), patients must overcome structural, clinical, and attitudinal barriers to trial enrollment. The goal of this systematic review was to study the relationship between socioeconomic status (SES), assessed either by direct or proxy measures, and CCT enrollment.

**Methods:**

The review team and medical librarian developed search strategies for each database to identify studies for this systematic review, which was conducted according to PRISMA guidelines. Inclusion criteria were as follows: studies published in relevant scientific journals between January 2000 and July 2022, primary sources, English literature, and studies conducted in the US. Sixteen studies fulfilled the inclusion criteria and were reviewed. The risk of bias assessment was conducted independently by two reviewers using the Newcastle Ottawa scale.

**Results:**

The initial search yielded 4070 citations, and 16 studies were included in our review. Four of the studies included used patient reported annual income as a measure of SES, while the remaining 12 studies used patient zip code as a proxy measurement of SES. Consistent with our hypothesis, 13 studies showed a positive association between high SES (patient‐reported or proxy measurement) and CCT enrollment. Two studies showed a negative association, and one study showed no relationship.

**Conclusions:**

The existing literature suggests that low SES is associated with lower participation in CCT. The small number of studies identified on this topic highlights the need for additional research on SES and other barriers to CCT participation.

## Abstract 2

A systematic review of the literature showed that socioeconomic status (SES) is a barrier to participation in clinical trials for cancer. However, proxy measures of SES were often used, such as median income in a given zip code, and further research is needed.

## 1 INTRODUCTION

Cancer clinical trial (CCT) enrollment at the initial course of treatment for oncology patients may increase survival rates, highlighting the importance of equitable access to CCTs.^1^ However, current CCT enrollment favors young, healthy, white patients treated at academic centers.^1^ Although CCTs present patients with access to novel treatments, fewer than 1 in 20 adult cancer patients enroll, raising concerns about generalizability of trial results.^2^ Low accrual rates have a negative impact on CCTs because they can prolong duration, delay analysis, and lead to early closure of studies.

Barriers to CCT participation have been studied at length, yet the rate of trial participation as not changed substantially over time.^3^ Furthermore, disparities in CCT enrollment for racial and ethnic minorities have increased over time.^4^ To increase enrollment and achieve equitable access to CCTs, patients must overcome structural, clinical, and attitudinal barriers to trial enrollment.^2^ A 2014 study found that the highest rated barriers to CCT participation included fear of side effects, insurance coverage concerns, fear of low efficacy, and fear of being randomized to placebo.^5^ This underscores the importance of addressing costs and benefits in the decision‐making process.

A patient's socioeconomic status (SES) reflects their relative position within a social hierarchy and their ability to consume resources. Studying the impact of SES on CCT enrollment is important because it can guide policy, advance treatment discovery, and increase survival. The goal of this systematic review is to study the relationship between SES and CCT enrollment. We hypothesized that low SES is a major barrier to participation in CCTs.

## 2 METHODS/LITERATURE SEARCH

We followed the PRISMA 2020 checklist. Our population of interest in this systematic review was patients with cancer who were offered enrollment in a clinical trial (CT). We excluded secondary sources, non‐English literature, and studies conducted outside of the US. This review was not registered. Since this review did not comprise human subjects research, we did not obtain IRB approval.

The review team and Clinical Informationist (JKN) developed search strategies for each database to identify studies for this systematic review. The search was restricted to studies published from 2000 to present and was conducted on July 27th, 2022. Of note, review articles were included in the search. Databases searched were PubMed, Embase, Cochrane, Web of Science, and Scopus. Limitations on the search were from January 1, 2000 to current. The search template (below) was translated into the individual databases with controlled vocabulary applied as applicable.

((“Clinical Trial*” AND (recruit* or enroll* or access* or barrier*)) OR “clinical trial recruit*” or “clinical trial enroll*” or “clinical trial access” or “cancer clinical trials”) AND (“Neoplasms” or “cancer*” or HNC or “head and neck cancer*” OR tumor* OR tumor* or osteosarcoma* OR oncolog* OR malignan* or nephroblastoma* OR metastatic OR metastasis OR metastases or neuroblastoma* or rhabdomyosarcoma* or hepatoma* or hepatoblastoma* or lymphoma* or leukemi* or leukaemi* or sarcom* or glioma* or astrocyte* or melanoma) AND (“Healthcare Disparities” or “Socioeconomic Factors” OR “Social Class” OR “Population Characteristics” OR “Economics” or “Socioeconomic*” OR “Population Characteristic*” OR “Economic*” or Sociodemographic* or “health services accessibility” OR “Health Services Access*”).

All article titles, abstracts, and full texts identified were reviewed by two reviewers through Emory University's subscription to Covidence, a screening and extraction tool for systematic reviews, to limit bias in inclusion. Disagreements were resolved by consensus. Since all identified articles were case–control or retrospective case studies, the risk of bias assessment was further evaluated independently by two reviewers (MWD and NCS) using the Newcastle Ottawa scale (Table S1).

## 3 RESULTS

The initial search yielded 4070 citations, and 16 of these studies were included in our review (Figure S1). Characteristics of included studies are summarized in Table 1.

**TABLE 1:** Summary of included studies.

Author	Study design	Sample size	Population	Measure of SES	Key findings
Abbas et al., 2022^40^	Case control	CT: 10, 518 Control: 2,225,730	Adults with gastrointestinal or hepatobiliary cancers	Zip code: median household income	Low‐income black patients were less likely to participate than high‐ or low‐income white individuals (*p* < 0.001).
Baquet et al., 2006^16^	Cross sectional	5154	Adults living in Maryland	Survey: annual income	Among respondents recruited to CT, Black (OR = 0.37) and middle‐income (OR = 0.57) respondents were less likely to enroll
					Increased levels of education and income determined the primary source for CT information: Respondents who were college graduates as well as those reporting incomes of $100,000 or more were more likely to receive information about CT from print media
					Residents from rural Western Maryland (OR = 0.46) and residents of rural Eastern Shore (OR = 0.30) were less likely to be recruited to CT
Baquet et al., 2008^41^	Case control	CT: 2240 Control: 96,906	Adults and children in Maryland with cancer	Zip code: Material deprivation summary score, social class summary score	For both female and male patients, the trend of low accrual onto trials with increasing deprivation scores was statistically significant (*p* < 0.001).
Behrendt et al., 2014^11^	Case control	1482	Women with breast cancer at Hope Comprehensive Cancer Center	Zip code: median household income	Accrual increases modestly with median household income (OR = 1.03, 95% CI 10.2–1.05 on multivariate analysis) Accrual is favorably associated with certain measures of socioeconomic disadvantage such as Medicaid eligibility and low educational attainment
Brierley et al., 2020^10^	Case control	CT: 449 Control: 1470	Adults with MDS	Zip code: total income/number of census returns	Patients in more affluent zip codes had a higher participation rate (*p* < 0.001). White race (*p* = 0.028) and reduced distance from trial site (*p* = 0.025) were significantly associated with increased trial participation
El Rayes et al., 2010^42^	Case control	CT: 118 Control: 6470, 1642	Patients with advanced pancreatic cancer in the Detroit tri county area	Zip code: median SES, census poverty status	CT participants were significantly less likely to reside in high poverty zip codes (*p* = 0.017). Women who resided within 3 miles of a research center were significantly more likely to enroll than individuals who live farther away (OR = 1.29)
Eskander et al., 2022^15^	Case control	CT: 1127 Control: 301,340	Pancreatic cancer	Zip code: median household income	CT enrollment associated with higher neighborhood income and education levels (*p* < 0.001) Living in the south as opposed to the northeast was associated with lower rates of CT enrollment (OR = 0.42)
Fayanju et al., 2020^12^	Case control	CT: 17,124 Control: 792, 719	Women with breast cancer	Zip code: median household income	Patients from highest area‐based income bracket (>63,000) were less likely to participate than those from the lowest income bracket (<38,000) and likelihood of enrollment declined with increasing income (*p* < 0.001)
Gross et al., 2005^43^	Case control	CT: 737 Control: 7384	Women >65 with breast cancer	Zip code: proportion below poverty level	CT participants were significantly less likely to reside in high poverty zip codes (*p* = 0.017) Women who resided within 3 miles of a research center were significantly more likely to enroll than individuals who live farther away (OR = 1.29)
Meyer et al., 2021^44^	Case control	20,053	Adults	Survey: annual income	Respondents in households earning more than $50,000 annually were 1.175 times as likely to participate in cancer CT even when controlling for other factors like race/ethnicity, sex, and age at diagnosis
Morshed et al., 2020^9^	Case control	988	Adult glioma patients at UCSF	Zip code: median household income, % below poverty level	Insurance type, employment status, median household income, and percent below poverty line were not associated with CT screening or enrollment for newly diagnosed glioma patients
Saphner et al., 2021^45^	Case control	CT: 72 Control: 39,196	Adults with cancer	Zip code: median household income, % below poverty level	Higher median household income, lower percentage of patients below poverty level, lower percentage of unemployment, and percentage college educated were significantly associated with a greater likelihood of participation in a trial on univariate analyses, but only male sex and advanced age were significant on multivariate analyses
Sateren et al., 2002^46^	Case control	CT: 24,332	Adults and children with cancer	Zip code: mean county income, mean poverty level	Areas with higher socioeconomic levels had significantly higher levels of CT accrual on univariate analysis, but high unemployment rate was the only significant surrogate measure of SES on multivariate analysis More than half of children aged 5–9 years are accrued to NCI sponsored CT compared with less than 1% of adults aged 75–79 years of age
Unger et al., 2016^23^	Prospective cohort	1591	Breast, lung, and colorectal cancer	Survey: household income	Patients with annual household income below $50,000 had a 32% lower odds of trial participation than higher income patients
Unger et al., 2013^22^	Cross sectional	5499	Adults with breast, lung, colorectal, and prostate cancer	Survey: annual income	Patients with lower income had lower CT participation (OR = 0.73) Concern about how to pay for CT participation was higher in low‐income patients (OR = 1.79) and lower education patients (OR = 1.50) Lower income predicted lower CT participation in patients >65, indicating an income disparity even in a population with universal access to Medicare
Winestone et al., 2019^8^	Case control	CT: 206 Control: 164	Children with AML	Zip code: proportion of residents below federal poverty line	Patients from zip codes with the lowest proportion of families in poverty were less likely to enroll than those from zip codes with higher concentrations of poverty (aRR = 0.70). This association varies by race/ethnicity

Historically, the study of SES and CCT enrollment has been hindered by a lack of data on income. While the measure of current income provides insight into a person's access to material goods and services, income is typically age dependent and does not include access to all assets such as wealth, insurance coverage, and disability benefits.^6^ In the absence of data on individual level income, area level income as a proxy measure of individual level SES is substituted.^7^

Four of the studies included in this systematic review used patient reported annual income as a measure of SES, while the remaining 12 studies used patient zip codes to develop proxy measurements of SES. Of the 12 studies that used proxy measurements for SES, 8 used area level income, 3 used area level poverty, and 1 used an area level material deprivation score considering the percentage of persons living in poverty, households without a car, unemployed persons aged 16 and older, and owner‐unoccupied housing. For studies that used income to measure SES, the cutoff for low SES ranged from a household income below $38,000–$50,000. For studies that used area poverty level as a proxy for SES, the cutoff for a high poverty level ranged from greater than 0.13%–4% of persons living in poverty.

Consistent with our hypothesis, 13 studies showed a positive association between high SES and CCT enrollment. Two studies showed a negative association, and one study showed no relationship. The results of these conflicting studies are further discussed below.

A 2019 study on CCT enrollment of children with acute myelogenous leukemia (AML) measured SES by calculating zip code‐based poverty. This study found that patients with low zip code‐based poverty rates (less than 4%) were less likely to enroll in AAML 1031 than patients who had higher zip‐based poverty rates, differing from previous literature which has shown that pediatric patients from low‐income areas have no differences in CCT enrollment.^8^ While pediatric CCTs are inherently different from adult CCTs, this study suggests that perhaps efforts to increase equity in pediatric CCTs may have been successful.

A 2020 adult glioma study found that minority race and SES did not impact CCT enrollment, rather patient location was the most significant predictor of trial enrollment for patients with newly diagnosed gliomas.^9^ This was consistent with another study in this review, which concluded that reduced distance from the trial site was associated with increased odds of trial participation.^10^

The 2020 adult glioma study also found that additional socioeconomic factors known to play a role in CCT enrollment for non‐glioma patient populations including employment status, insurance type, household income, and percent below poverty line were not associated with trial enrollment for glioma patients.^9^ This contrasts with a 2014 breast cancer study that found accrual is favorably associated with certain measures of socioeconomic disadvantage such as Medicaid eligibility and low educational attainment independent of income.^11^

The study by Fayanju and colleagues on CCT participation among breast cancer patients found that trial enrollment for the 12‐year study period was associated with white race, higher levels of education, and lower levels of area‐based income.^12^ Over the course of the study period, overall trial participation declined sharply, reflecting the trend to smaller, and fewer trials over time. This study is unique in demonstrating complex and varying interactions between income, race, and trial availability over 12 years. At the beginning of the study period, high‐income Asian/Pacific Islander (API), Hispanic, and white patients had higher rates of participation than their low‐income counterparts. By the end of the study, low‐income API and Hispanic patients were more likely to participate than their high‐income counterparts. The likelihood of trial participation among all racial/ethnic groups has decreased over time, but gains among low‐income API, Hispanic, and white patients appear to have occurred in proportion to their high‐income counterparts, suggesting that efforts to diversify trial enrollment have been effective.

## 4 DISCUSSION

Expanding CCT enrollment is advantageous for patients, researchers, and future generations, given it's crucial role in driving breakthroughs in cancer treatment discovery. Although CCTs provide patients with access to novel treatments, close monitoring, and increased survival,^1^ enrollment among adults remains low. Generalizability is important to consider when applying trial results to real world patients. A lack of socioeconomic diversity in CTs limits generalizability because SES influences health both directly and indirectly through social, environmental, and medical factors such as chronic stressors, dietary options, and housing quality, and pollutants.^13^

To our knowledge, this is the first attempt at a systematic review to assess the relationship between SES and CCT enrollment. Consistent with our hypothesis, most included studies (13/16) concluded that lower SES is associated with decreased rates of CCT enrollment. While this systematic review does not explain why this trend exists, it suggests that low SES is associated with barriers that play a significant role in CCT enrollment.

The findings in our study were expected, but we were surprised to see that most studies used proxy measures of SES, most commonly zip code, rather than direct measures of SES. Although zip code may be an adequate proxy measure when individual or household SES data are not available, direct measures of SES are preferred.^14^

Multiple studies included in this systematic review found that lower rates of CCT enrollment were associated with increased distance from the clinical site, lower education levels, and minority race/ethnicity. Eskander et al.^15^ concluded that geography remains a significant barrier to CCT access: Participants in that study who lived in the south needed to travel twice as far and had half the odds of enrolling in a CCT when compared to their northeastern counterparts. Baquet et al.^16^ studied CCT recruitment and participation for adult Marylanders and found that respondents of rural Western Maryland and the rural Eastern Shore were significantly less likely to be recruited to CCT. These findings are consistent with data on growing health disparities that exist between rural and urban communities, with rural communities having lower life expectancy and higher mortality rates from most top causes of death.^17^ The effects of rural versus urban location on CCT enrollment are likely to be underestimated in the included studies, considering neighborhood level measures of SES inadequately account for individual SES in rural settings.^18^ Seventeen percent of the US population lives in rural or remote communities, but only 9% of physicians practice in rural areas.^19^ Additionally, rural communities have higher poverty rates and lower educational attainment when compared to urban communities.^19^

The role of race and ethnicity in CCT enrollment has been an area of frequent study, yet racial/ethnic minorities are still not adequately represented in CCT. Behrendt et al.^11^ discovered that adjustment of SES helped reveal ancestry related disparities that would otherwise remain obscure, demonstrating that studying the intersection between race/ethnicity and SES in addition other vulnerabilities is key to understanding disparities and achieving equity in CCT enrollment.

When examining the relationship between SES and CCT enrollment, it is important to acknowledge the relationship between SES and race. Researchers have long assumed that racial differences in SES substantially contribute to racial disparities in health. However, complex patterns emerge when studying the relationship between race, SES, and various health outcomes.

In the US, racial groups capture differences in power, status, and resources because the structure of segregation and its consequences have remained relatively intact over time.^20^ Strikingly high levels of racial inequality in SES remain today, contrasting with the perception that racial inequalities have narrowed over time.^21^ Poverty varies greatly by race, with black and Hispanic levels of poverty being two to three times higher than that of whites.^21^ Even after controlling for SES, racial disparities in health often remain, with white males and females living 2–4 years longer than their black peers at every level of income.^21^ While this paper does not aim to study the relationship between race, SES, and CCT enrollment, it is important to acknowledge the co‐founding concerns given overlapping populations.

In a study by Unger and colleagues on patient income level and CCT participation, lower income predicted lower CCT participation in patients age ≥ 65, indicating an income disparity even in a population with universal access to Medicare.^22^ This finding suggests that while the cost of a CCT itself may not be a barrier to enrollment, other structural and attitudinal factors may play a significant role in the decision to enroll. Lower income patients are more likely than higher income patients to be concerned about how to pay for the nonmedical costs of CCT participation, which is consistent with the idea that low SES is a barrier to CCT enrollment.^23^

Cancer is the most expensive chronic disease in the United States and the cost of care has increasingly shifted to patients in the form of premiums, copays, coinsurance, and deductibles leading to financial toxicity.^24^ In addition to direct costs, loss of wages also contributes to financial burden, with one study reporting that approximately one third of patient annual wages were lost within 1 year of an early stage cancer diagnosis.^25^, ^26^ Financial toxicity is defined as the objective and subjective patient‐level impact of the costs of cancer care and can afflict individuals—even those with health insurance—from any socioeconomic background.^24^, ^27^ Financial toxicity is a recognized barrier to CT enrollment,^24^, ^27^, ^28^, ^29^ with indirect costs exacerbating obstacles to enrollment.^28^, ^30^, ^31^, ^32^ Patients enrolled in CTs are subject to additional expenses compared to their non‐enrolled peers, including the costs of more frequent clinical visits and travel to trial sites.^24^, ^33^, ^34^, ^35^ The Affordable Care Act (ACA) requires coverage of routine care costs for patients enrolled in approved CTs, with exceptions for insurance programs predating the ACA; however, coverage of additional and indirect costs is not required. The majority of out of pocket costs associated with CT enrollment are nonmedical costs, such as travel and lodging.^28^, ^30^, ^31^, ^32^, ^36^, ^37^ Indeed, patients enrolled in CTs spend over $600 per month out of pocket for nonmedical costs alone, with one out of five patients spending at least $1500 per month.^36^ These high out of pocket expenditures disproportionately effect non‐white and Latinx/Hispanic patients^36^ and may represent one factor contributing to the lack of diversity in CTs. Implementation of programs that address financial toxicity through a multipronged approach that includes financial reimbursement, increased accessibility, and patient advocacy may increase CT enrollment, particularly among patients with lower SES.

Although we found only one study in our review involving children, it suggests that SES may have different effects on CCT enrollment in children versus adults. Most children with cancer in the United States are enrolled in CCTs, in part because CCTs are considered the standard of care.^38^ The Children's Cancer Group (CCG), a cooperative CT group, was established in 1955 to address poor pediatric cancer outcomes.^39^ In 2000, four large cooperative CT groups merged to create the Children's Oncology Group (COG), which is estimated to treat 90% of children with cancer in the United States today.^39^ Since the implementation of cooperative CT groups, pediatric cancer survival has gone from 30% in the 1960s to over 80% today.^38^ The improvement in pediatric oncology survival parallels improvements in multidisciplinary care and research, mediated by cooperative CT groups.^39^ Additionally, free housing and financial assistance provided to pediatric oncology patients through programs such as such as Ronald McDonald House and St. Jude may have a unique influence on the pediatric CCT participation.

Our study has several limitations. Data on income is often not available in large data sets, and as a result, most studies included in this systematic review use proxy measures of SES based on zip code. Due to heterogeneity in study methodology, we were not able to conduct a meta‐analysis. Despite these limitations, this systematic review highlights the importance of considering SES along with additional barriers to CCT enrollment. The surprisingly small number of studies also highlights the need for additional research on SES, other patient‐level barriers, and strategies for equitable enrollment.

## 5 CONCLUSIONS

SES is a major barrier to enrollment in CTs for cancer, at least in a subset of patients. Further research is needed on the role of SES in CT enrollment for different cancers, ideally with more direct (rather than surrogate) measures of SES.

## AUTHOR CONTRIBUTIONS

**Maja Wichhart Donzo:** Conceptualization (equal); data curation (equal); investigation (lead); methodology (equal); writing – original draft (lead); writing – review and editing (equal). **Grace Nguyen:** Data curation (equal). **John Nemeth:** Conceptualization (equal); data curation (equal); methodology (lead); writing – review and editing (equal). **Maryanna Owoc:** Writing – original draft (equal); writing – review and editing (equal). **Leila Mady:** Writing – original draft (equal); writing – review and editing (equal). **Amy Chen:** Conceptualization (equal); supervision (equal); writing – original draft (equal); writing – review and editing (equal). **Nicole C. Schmitt:** Conceptualization (equal); data curation (equal); investigation (equal); methodology (equal); supervision (lead); writing – original draft (supporting); writing – review and editing (lead).

## DISCLOSURE STATEMENTS

Schmitt—*Advisory Board*: Checkpoint Surgical; *Book Royalties*: Plural Publishing, *Consulting*: Sensorion; *Research Funding*: Astex Pharmaceuticals.

## Supporting information


Figure S1.

## Data Availability

No new data were generated or analyzed in this manuscript.
