# Supplementary material for: Effects of socioeconomic status on enrollment in clinical trials for cancer: A systematic review
Source: Cancer Med. 2024 Jan 3;13(1):e6905. doi: 10.1002/cam4.6905 (PMC10807561; doi:10.1002/cam4.6905)
Supplement: Supplementary file 1 — Figure S1. [file CAM4-13-e6905-s001.pdf]

Supplemental Data

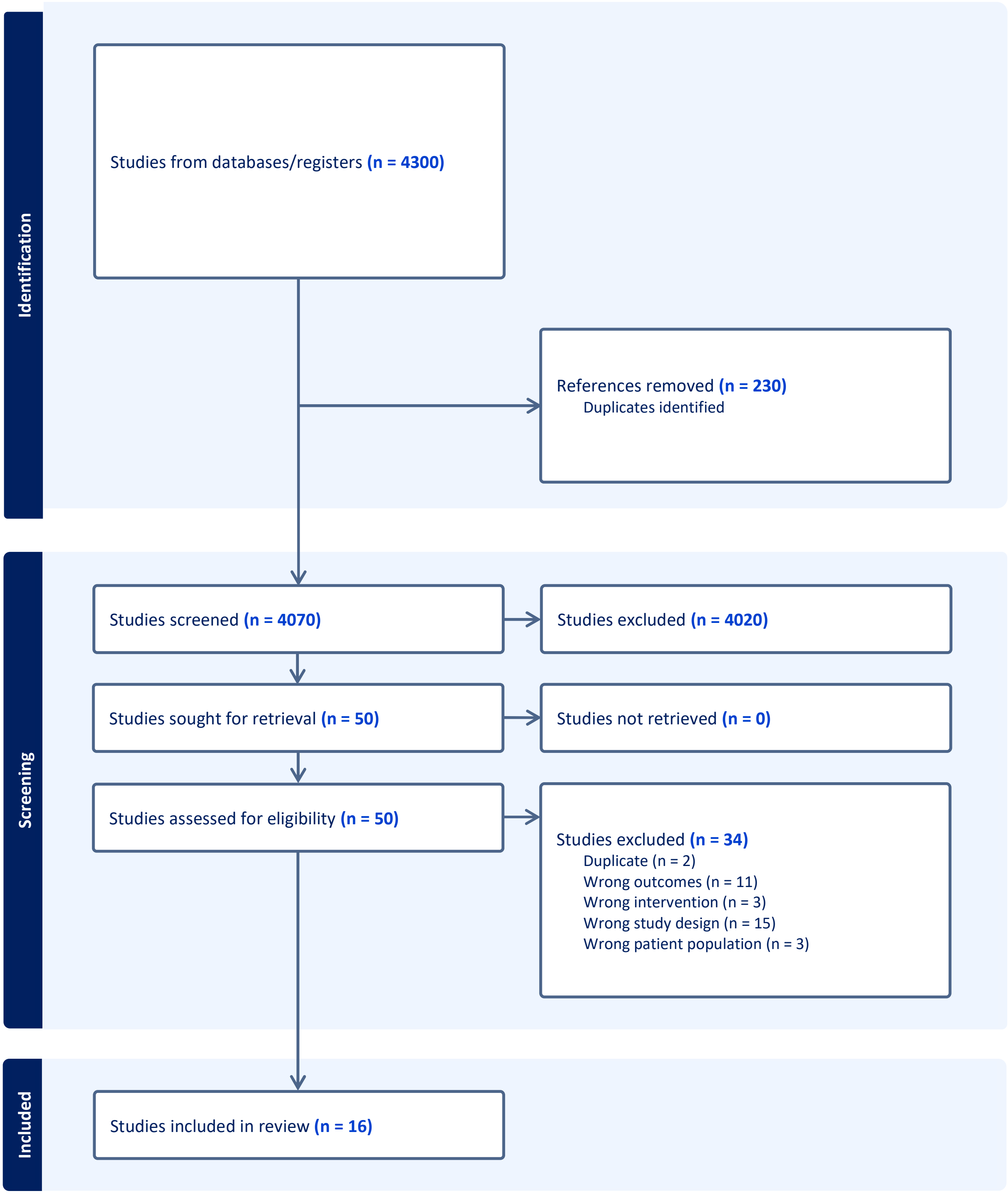

Supplemental Figure S1: PRISMA flow diagram of study selection.

Author	Reviewer 1	Reviewer 2
Abbas et al., 2022	7	8
Baquet et at., 2006	7	8
Baquet et al., 2008	8	8
Behrendt et al., 2014	8	9
Brierley et al., 2020	8	9
El Rayes et al., 2010	7	7
Eskander et al., 2022	8	9
Fayanju et al., 2020	8	8
Gross et al., 2005	8	9
Meyer et al., 2021	7	8
Morshed et al., 2020	8	9
Saphner et al., 2021	8	9
Sateren et al., 2002	6	5
Unger et al., 2016	8	8
Unger et al., 2013	8	8
Winestone et al., 2019	7	8

Table S1: Newcastle-Ottawa Quality Scoring (maximum score = 9)
